# Effects of Subthalamic Nucleus Deep Brain Stimulation on Facial Emotion Recognition in Parkinson's Disease: A Critical Literature Review

**DOI:** 10.1155/2020/4329297

**Published:** 2020-07-17

**Authors:** S. Kalampokini, E. Lyros, P. Lochner, K. Fassbender, M. M. Unger

**Affiliations:** Department of Neurology, University Hospital of Saarland, Kirrberger Straße, 66421 Homburg, Germany

## Abstract

Deep brain stimulation (DBS) of the subthalamic nucleus (STN) is an effective therapy for Parkinson's disease (PD). Nevertheless, DBS has been associated with certain nonmotor, neuropsychiatric effects such as worsening of emotion recognition from facial expressions. In order to investigate facial emotion recognition (FER) after STN DBS, we conducted a literature search of the electronic databases MEDLINE and Web of science. In this review, we analyze studies assessing FER after STN DBS in PD patients and summarize the current knowledge of the effects of STN DBS on FER. The majority of studies, which had clinical and methodological heterogeneity, showed that FER is worsening after STN DBS in PD patients, particularly for negative emotions (sadness, fear, anger, and tendency for disgust). FER worsening after STN DBS can be attributed to the functional role of the STN in limbic circuits and the interference of STN stimulation with neural networks involved in FER, including the connections of the STN with the limbic part of the basal ganglia and pre- and frontal areas. These outcomes improve our understanding of the role of the STN in the integration of motor, cognitive, and emotional aspects of behaviour in the growing field of affective neuroscience. Further studies using standardized neuropsychological measures of FER assessment and including larger cohorts are needed, in order to draw definite conclusions about the effect of STN DBS on emotional recognition and its impact on patients' quality of life.

## 1. Introduction

Deep brain stimulation (DBS) has evolved into one of the most effective established therapies for the treatment of movement disorders, with subthalamic nucleus (STN) being a major target for Parkinson's disease (PD) [1, 2]. DBS, with a high-frequency electrical stimulation (>100 Hz) of specific brain targets, mimics the functional effects of a lesion. High-frequency stimulation exerts an inhibitory effect on neuronal activity; proposed mechanisms are the masking of encoded information by imposing a high-frequency pattern [3], suppression of abnormal beta oscillations [4, 5], stimulation of inhibitory gamma-aminobutyric acid (GABAergic) afferents to the target nucleus [6] or other efferent projections or passing fibres [7], and lastly the inhibition of production or release of neurotransmitters and hormones [8]. Nevertheless, it has become clear that the mechanisms involved in DBS are more complex, as neural elements may be excited or inhibited, reaching novel dynamic states of equilibrium and developing various forms of neural plasticity [9].

The basal ganglia are part of cortico-subcortical networks involved in the selection (facilitation or inhibition) of not only movements but also behaviours, emotions, and thoughts. STN, located at the diencephalic-mesencephalic junction, has a central position in the corticobasal ganglia-thalamocortical circuits, each of which has sensorimotor, associative, and limbic functions [10]. STN can be functionally divided into sensorimotor (dorsolateral), limbic (medial), and cognitive-associative (ventromedial) areas [11]. The STN is not only a relay station controlling thalamocortical excitability (the so-called “indirect” pathway of the basal ganglia circuit) [11, 12] but also an important input regulatory nucleus of the basal ganglia, receiving projections from the frontal cortex (the so-called hyperdirect pathway [13, 14]), thalamus, and brainstem. Indeed, the contribution of STN to nonmotor, especially limbic, functions has attracted increasing attention based on the results of animal studies [15–18] as well as studies of PD patients receiving high-frequency stimulation [19–22].

STN DBS has proven beneficial effects on different motor symptoms of the disease (particularly tremor, rigidity, motor fluctuations, and levodopa-induced dyskinesias) [23, 24], which seem to be long-lasting [25]. Additionally, it allows a significant reduction (in the range of 50 to 60%) of dopaminergic medication postoperatively [24, 26]. There is also evidence that STN DBS reduces anxiety, pain, and nonmotor fluctuations [27] and improves sleep and generally patients' quality of life [23, 28]. Nevertheless, adverse effects on some neuropsychiatric, cognitive, and behavioural symptoms following STN DBS have been reported such as increased apathy [27, 29], impulsivity [27], hypomania [30, 31], and even attempted or completed suicide [22, 32]. STN DBS may also result in worsening of memory and overall cognition [33, 34], processing speed [33], attention [33], verbal fluency [33–35], and executive functions [33–35]. These adverse effects occur particularly in PD patients with preexisting cognitive [27] or behavioural symptoms [23, 36, 37] as well as older patients (≥70 years), patients with high dopaminergic treatment, reduced levodopa response and axial signs such as postural instability and freezing of gait or dysarthria [38, 39].

Among neuropsychiatric symptoms of PD, facial emotion recognition (FER) has also been reported to change after STN DBS. Yet, the results of studies concerning FER after STN DBS are inconsistent [40–49]. The ability to recognize emotions in others' facial expressions is an essential component for nonverbal communication and social interactions [50]. In fact, impaired FER can lead to poor social integration and difficulties in interpersonal relationships such as the feeling of frustration and this of social isolation [51], which is linked to poorer mental health and quality of life [52, 53]. Deficits in interpreting social and emotional cues can affect PD patients' social behaviour and have implications for living with family members or caregivers [54].

## 2. Methods

**Figure 1:**
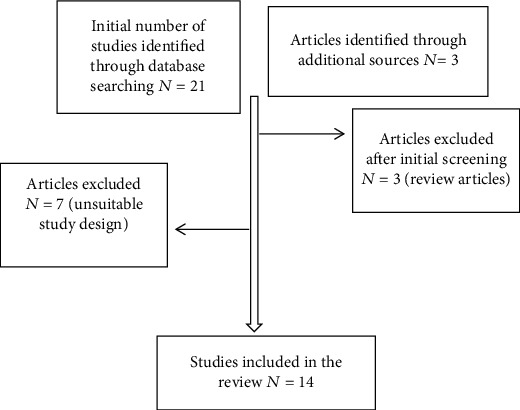
Flow diagram of studies assessed for the review.

In order to further investigate the issue of FER after STN DBS, we conducted a literature search of the electronic databases MEDLINE and Web of science between 2000 and 2019 for studies published in English language. The key search terms were as follows: facial emotion recognition, Parkinson's disease, subthalamic nucleus, and deep brain stimulation. The inclusion criteria were (1) studies assessing emotion recognition from facial stimuli in PD patients undergoing STN DBS and (2) studies providing data in different conditions (pre- or postoperative and ON or OFF stimulation). The exclusion criteria were (1) review articles and (2) unsuitable study design or stimuli, e.g., affective pictures, films, and vocal stimuli. The search was implemented by manual search of the references of the identified studies. The search yielded 24 studies, from which 10 were excluded, resulting in a total of 14 studies, which were included in the review. A flow chart of studies assessed for this review can be seen in Figure 1. The data that were extracted from the included studies were as follows: authors' name, year of publication, sample size, patients' characteristics (sex, age, duration, and severity of disease), FER test (number of stimuli and emotions and display time), levodopa equivalent dose before and after STN DBS, assessment conditions (stimulation ON or OFF and medication on or off), assessment time point after STN DBS, and outcome on FER performance (response accuracy and reaction time). Quality assessment of studies was done using the Methodological Index for Non-randomized Studies (MINORS) [55], which was greater than 10 in all included studies indicating a good quality. In this review, we discuss the discrepancies between studies and the mechanisms through which STN DBS can affect FER in PD patients.

## 3. Results

### 3.1. Studies Assessing Facial Emotion Recognition after STN DBS

A few studies assessed recognition of emotional facial expressions after STN DBS with relatively inconsistent findings. The characteristics of studies assessing FER in PD patients undergone STN DBS are summarized in Table 1. In the recent meta-analysis of Coundouris et al. [56] examining social perceptual function in PD, the subanalysis concerning DBS showed that PD patients were significantly impaired in perception functions after STN DBS surgery from either facial or vocal stimuli compared to matched healthy controls (HC). The majority of studies included PD patients eligible for DBS according to standard inclusion and exclusion criteria [57], i.e., patients with idiopathic PD and severe motor disability, clear response to levodopa, occurrence of disabling levodopa-related motor complications and absence of dementia, significant neuropsychiatric disorders, and abnormalities on brain MRI. All patients underwent bilateral STN DBS. The HC included in some studies had no history of neurological disease, brain injury, or dementia and were most commonly matched for age, gender, and education with the PD patients. A variety of facial stimuli was used in the studies with the most common ones the Ekman and Friesen series [58], the Hess and Blairy series [59], the Nim Stim Set [60], and the Karolinska directed emotional faces database [61]. Moreover, most studies included various background neuropsychological testing with most common global cognitive measures (such as the Mini mental state examination and Mattis dementia rating scale), semantic and phonemic verbal fluency tasks, and executive function testing such as the Stroop test, the trail making test, and the Wisconsin card sorting test, while only a few used visuospatial tests [40, 46, 62] and the Benton facial recognition test [41, 42, 44, 46, 49, 62, 63].

Regarding the methodology of studies conducted so far, patients were tested in alternating experimental settings with stimulation ON or OFF and medication on or off, i.e., DBS ON/med on, DBS ON/med off, DBS OFF/med on, and DBS OFF/med off. Studies have either compared the pre- to postoperative condition after STN DBS within the same PD group [40–42, 63, 64], matched PD groups [44, 48, 49], or PD patients with matched HC [46, 48, 62, 65]. Most studies reported impaired FER after STN DBS compared to before surgery [40–44]. Predominately, the recognition of negative emotions worsened after DBS [40, 43, 44, 63]. Yet, others failed to show a significant change of FER after surgery [46–49]. One study [62] reported that the combined effects of DBS and L-dopa were beneficial for recognition of emotional facial expressions. Additionally, a few studies compared the ON versus OFF DBS stimulation condition postoperative in PD patients [43, 45, 46, 62]. Aiello et al. [46] and Mondillon et al. [62] showed no significant difference in FER after STN DBS with the stimulator either ON or OFF as long as the patients were on medication. In the off medication state, PD patients exhibited a worse FER recognition in the ON stimulation condition as opposed to OFF [62]. Moreover, Geday et al. [66] reported that STN stimulation affected the general perception of facial expressions; i.e., these were scored as less pleasant in the ON condition as opposed to OFF. Lastly, Wagenbreth et al. [45] in a recent study assessed postoperative PD patients in an explicit emotional processing task, where the patients had to name the emotional status depicted in the eye region, and showed a general decrease in response accuracies under STN DBS in the ON condition compared to the OFF condition.

Regarding the recognition of specific emotions (i.e., the seven basic emotions: happiness, surprise, fear, anger, sadness, disgust, and neutral), few studies showed a significant reduction of decoding accuracy for sadness [40, 41, 63], fear [41, 42, 44, 63], anger [40], and a trend for disgust [40] after DBS compared to before, although there was not always a comparison with a HC group before surgery. Moreover, Enrici et al. [48] showed a significant impairment of FER for surprise in the STN-DBS-PD group compared to the HC group. With regard to specific emotion performances in different stimulation conditions, Schroeder et al. [43] showed impaired anger recognition in PD patients in the ON STN condition compared to the OFF condition, while Mondillon et al. [62] found a significant decrease in the recognition of disgust ON STN stimulation and a tendency toward impaired recognition of fear OFF stimulation compared to HC (both off medication). Aiello et al. [46] reported that in the OFF condition soon after surgery (5^th^ postoperative day), patients were impaired in recognizing sadness, while few months after (2-6 months) and with the stimulator ON, they exhibited impaired disgust recognition compared to HC (which was also evident preoperative). Furthermore, Wagenbreth et al. [45] showed that ON condition of STN DBS worsened the explicit processing for disgust stimulus material (eye region and words) but improved the explicit processing of fear stimuli compared to the OFF condition. In contrast, Biseul et al. [44] showed that a deficit in recognition of fear (compared to the pre-operative state and HC) was identical in the PD patients with the stimulator either ON or OFF.

## 4. Discrepancies between Studies

### 4.1. Methodological Differences of Studies

Most studies assessing FER after STN DBS had small sample sizes (<30) except for one [47]. Furthermore, the majority of studies had as outcome the accuracy score in FER tasks, without additionally measuring the reaction time of the participants' responses, which in the case of prolongation would also indicate impairment. The reasons for discrepancies between studies could be methodological, concerning study design, i.e., FER task, testing conditions, or time of assessment. With regard to FER tasks, they varied in terms of stimulus material used. The majority of studies [41, 42, 44, 63] used facial stimuli from the Ekman and Friesen series in its original black and white version [58]. Nevertheless, two studies used computer transformed stimuli with intensities of two emotions [43] or intermediate expressions differing in emotional intensity [40]. Other studies used stimuli containing the region around the eyes as well [45, 49]. Less commonly, authors used facial stimuli in color such as the Karolinska directed emotional faces database [67] or Nim Stim Set [60]. The methodological drawbacks of FER tasks should also be born in mind; most tasks use static facial expressions, categorization, and forced choice tasks (naming of emotional faces), which are less sensitive than visual analog scales, mainly because of categorization biases [68]. The patients have to select the appropriate label among the choices that are mostly negative, so the probability of an incorrect response is higher for the negative emotions. Moreover, low-intensity facial stimuli are associated with worse FER performance [52]. The studies included in our review did not test for different intensities of stimuli except for one [40], which showed FER worsening after surgery irrespective of stimuli intensity. The number of stimuli also varied across studies. Another factor is the time given to patients to select the appropriate answer, which was variable among studies as well. In case of no time limit, it is possible that patients recruit other perceptual strategies [69, 70]. With regard to this, Mondillon et al. [62] used a rapid representation design, which may correspond more properly to the microexpressions encountered in everyday life [71].

The follow-up periods after STN DBS also varied ranging from days to 48 months after surgery. In fact, some studies testing FER relatively soon after surgery (3 months) [40–42, 63] found a worsening of FER, whereas the few studies assessing FER later on (one year after surgery) [47, 48] did not. It can be argued that the histological changes after DBS surgery evolve with time as neuronal plasticity develops [9], which makes the interpretation of the results of studies with different assessment times after surgery challenging. Moreover, differences of patients' characteristics might at least partly account for the discrepancies between studies. Although patients' age, disease duration, and general cognitive measures were comparable among studies, subtle cognitive or affective differences might have been present. Moreover, the mean Hoehn and Yahr score was ≤2 in most studies on medication [41, 42, 48, 49, 63], whereas few studies either did not report the score [43–46, 62] or reported it off medication [40]. Despite the fact that most studies included patients according to standard DBS selection criteria [57], others recruited early PD patients [49] or used additional criteria such as a certain motor response to DBS or the absence of a dysexecutive syndrome [62].

### 4.2. Clinical Factors: Influence of Electrode Positioning, Stimulation, and Disease on Facial Emotion Recognition Changes after STN DBS

Most FER studies verified accurate DBS electrode placement using imaging techniques, intraoperative microelectrode recordings and macroelectrode stimulation, while only a few studies reported additional confirmation of the electrode positioning by MRI postoperatively [43, 48, 62, 66]. However, studies did not report FER outcomes in relation to the exact localization of DBS electrodes and active contacts, which can be reconstructed using specialized software based on postoperative imaging. Variable electrode positioning after STN DBS is thus a factor that could possibly have accounted for discrepancies of observed results. Another important issue is how to distinguish the effects induced by surgery from those induced by STN stimulation. A few studies addressed this issue by comparing the test scores with stimulation “ON” versus “OFF” [43, 62, 66]. The OFF stimulation assessment is done one hour after turning the stimulator off; however even then, there are effects of stimulation present, meaning that it is not a complete “OFF” condition. This time corresponds to the time until most of the motor symptoms reappear [72], but it is unclear what happens with the nonmotor effects. Moreover, the same applies to the long-lasting neural reorganization following STN stimulation [9], which cannot be eliminated by merely turning the stimulator OFF [54]. Additionally, contact configuration (bipolar or monopolar) and stimulation parameters, including frequency, pulse width, and especially stimulation intensity, varied between patients among studies resulting in the variable volume of nucleus tissue stimulated and thus variable nonmotor and emotional effects [73, 74]. Indeed, altering stimulation parameters can often lessen the stimulation-induced behavioural problems [75]. In this respect, only half of the studies reported the stimulation parameters of PD patients [41–43, 45, 46, 49], which were selected based on patients' optimal motor effect.

Another issue is whether PD patients with normal FER performance before and deficit after DBS actually had a subtle FER deficit before DBS being revealed after surgery. Indeed, PD patients exhibit significant social perceptual deficits including FER impairment [56, 76]. Areas involved in the process of recognizing emotions in faces such as the amygdala, basal ganglia, insula, the orbitofrontal, and anterior cingulate cortex are affected by PD-related pathology [77]. Not all studies examined the presence of a FER deficit before surgery by comparing with HC. For example, PD patients in the study of Aiello et al. [46] had a FER impairment (for disgust, on medication) compared to HC even before DBS, unlike other studies. With regard to whether FER impairment after STN DBS is due to the disease's natural progression [78] or rather an effect of DBS, studies showed a FER deficit already three months after DBS in PD patients who had an intact FER prior to surgery [40–42]. Moreover, McIntosh et al. [49], who recruited early PD patients randomized in two PD groups (optimal drug therapy or optimal drug therapy and DBS), used various affective tasks including few facial emotional stimuli and found an impairment of emotion assessment in PD patients as opposed to healthy participants but no difference irrespective of treatment type or treatment state (ON, OFF).

## 5. How STN DBS Can Affect Facial Emotion Recognition in PD

### 5.1. The Limbic Role of STN

A large number of structures including the orbitofrontal cortex, the anterior cingulate cortex, the amygdala, the right parietal cortex and visual processing areas like the occipitotemporal cortex participate in multiple processes and at various points in time in the recognition of emotions in faces [79, 80]. Moreover, neural substrates responsible for FER involve the basal ganglia limbic loop [81]. The STN can be considered part of a widely distributed neural network involved in FER either through processing limbic, i.e., emotional and associative information within the nucleus itself, or through its impact on other subcortical and cortical limbic areas. The limbic part of the STN is partly reciprocally connected with limbic parts of the basal ganglia [82, 83] such as the ventral striatum [84, 85] and ventral pallidum [11], the major output region of the limbic circuit [81]. There are also efferents from the STN to the substantia nigra, mostly to the pars reticulata [86] responsible for the regulation of dopamine release [11, 87], pedunculopontine nucleus [88], and amygdala [89, 90]. Additionally, the medial (limbic) tip of the STN projects to the ventral tegmental area, from which the mesolimbic dopaminergic pathway originates, involved in mediating primary motivational behaviours [11]. STN is also part of the indirect pathway connecting the striatum and internal globus pallidus, which is considered the “stop” or “no-go” pathway reducing thalamic and cortical activity [91]. Furthermore, STN receives input directly from the cortex through the hyperdirect pathway [14] and particularly from the frontal and prefrontal areas such as the anterior cingulate cortex [13, 92] and the orbitofrontal cortex [90, 93], which participate in the recognition of emotions in faces [79, 80].

Indeed, various studies support the involvement of STN in limbic functions. Vicente et al. [94] reported that STN stimulation affects the subjective experience of emotion, and Serranova et al. [95] showed that aversive stimuli were scored as more unpleasant with STN DBS ON compared to OFF. Conversely, in the study of Schneider et al. [96], stimulation (ON) had a positive mood induction effect and improved emotional memory. Neurophysiological studies support the limbic role of STN as well. Kühn et al. [97] showed a modulation of STN local field potential alpha activity a few days after DBS on medication in response to emotionally arousing pictures (irrespective of valence, i.e., direction of behavioural activation away from unpleasant or towards pleasant stimuli). In contrast, Brücke et al. [98] and Huebl et al. [99] found a significant modulation of STN alpha activity with emotionally arousing pictures, which correlated with the valence but not the arousal, i.e., intensity of the emotional activation [98]. With regard to this, Sieger et al. [100] showed that the activity of some STN neurons was related to emotional valence, whereas the activity of different neurons responded to arousal. Moreover, functional neuroimaging studies support the STN involvement in emotional processes, for example, when viewing emotion-inducing short film excerpts (such as disgust, amusement, and sexual arousal [101]) or pictures of beloved persons (maternal and romantic love [102]).

Therefore, the changes in emotional processing tasks after STN DBS such as the worsening in FER might be attributed to a direct effect of DBS on STN or disruption of its connections with the other basal ganglia or cortical areas involved in FER after surgery. Interestingly, STN DBS may modulate neural functions in different ways including both short- and long-term mechanisms of neuroplasticity [89]. Peron et al. [73] suggest that STN DBS might bring instability into the basal ganglia system, which synchronizes the neural activity of distinct areas involved in FER such as the orbitofrontal cortex and the amygdala [42] or recognition of facial stimuli such as the fusiform area [66]. Haegelen et al. [103] suggest that the inhibition of the STN by DBS would lead to failure of transmission of cortical information to limbic areas such as substantia nigra pars reticulata and the ventral tegmental area, which are additionally affected by dopaminergic loss in PD. Another hypothesis based on Graybiel's model [104] is that the basal ganglia and in particular the limbic circuit including STN select emotional patterns without conscious control (just like they select motor patterns) based on their connections with cortical and subcortical areas. STN DBS would disrupt this coordination process and lead to misinterpretation of emotional stimuli. Another mechanism that explains how STN DBS may result in FER worsening is the modulation of STN oscillatory activity [4, 5, 105, 106]. Indeed, there is an emerging role of low-frequency alpha- and beta-oscillations in the STN in PD, which are not exclusively motor [107] and seem to be involved in limbic and emotional information processing [108]. In fact, STN areas involved in the origin of beta activity project not only to sensorimotor areas but also to areas associated with cognitive, behavioural, and emotional functions such as prefrontal, frontal, higher order sensory, and temporal areas [107].

### 5.2. Changes in Cerebral Metabolism after STN DBS

Neuroimaging studies have shown changes in glucose metabolism or regional blood flow after STN DBS in areas associated with facial emotion processing. Indeed, many PET studies showed a decrease in resting state-metabolism post-DBS (in the ON condition) in precentral, frontal areas such as the anterior cingulate gyrus [109–111] and temporal areas [42, 110]. Contrarily, other studies found a significant increase in regional cerebral metabolism at rest after STN DBS in limbic and associative projection territories of the basal ganglia such as the prefrontal [112, 113], frontal, and anterior cingulate cortices [66, 113, 114] as well as temporal and parietal cortex [115]. Interestingly, Le Jeune et al. [42] reported a positive correlation between impairment of fear recognition and glucose metabolism changes in the right orbitofrontal cortex. Hence, STN DBS may induce modifications in the striato-thalamo-cortical circuits involving the orbitofrontal and anterior cingulate cortex or modulate a frontal network connected to the limbic and associative STN territories. Moreover, Le Jeune et al. [42] showed an increase in the activation of the right fusiform gyrus after STN DBS (in the ON condition), whereas Geday et al. [66] found a reduced activation (off medication) when PD patients viewed emotional faces (as opposed to neutral faces) compared to HC. Based on these observations, the difficulty of PD patients to decode emotions after STN DBS might be attributed to the inhibition of the activity in the fusiform gyrus, which is normally induced by emotional visual stimuli and particularly facial stimuli [116, 117], or in a network including the fusiform gyrus and the STN [66]. Other neuroimaging studies [42, 63] suggested that STN DBS may also modify the activity of amygdala, a key structure for FER, which has also connections with the orbitofrontal and anterior cingulate cortices [118]. Indeed, STN, particularly its anterior-ventral part, is functionally connected with medial temporal structures including the hippocampus and amygdala [89, 90, 107]. Furthermore, a part of the ventral amygdalofugal pathway, one of the main efferent pathways of the amygdala, passes close to (through and around) the STN [89] and might be affected from surgery.

### 5.3. Role of Neurotransmitters in Facial Emotion Recognition after STN DBS

Another widely discussed issue is the contribution of reduction of dopaminergic therapy after DBS to FER impairment. Gray and Tickle-Degnen [76] in their meta-analysis reported that emotion recognition impairment of PD patients was greater, although not significantly, in the hypodopaminergic state compared to the medicated state, consistent with the assumed role of dopamine in emotion regulation [119]. In contrast, Coundouris et al. [56] in their meta-analysis showed that medicated PD patients had significantly greater social perceptual deficits than nonmedicated PD patients, which might be due to the dopaminergic overdose of regions involved in social perception, relatively intact from dopaminergic denervation [56]. Dopamine might therefore have beneficial effects on FER rather in the advanced stages as opposed to the early stages in which the mesocorticolimbic pathways are relatively spared [52]. Furthermore, the dopaminergic loss in PD varies and progresses in different ways in the affected areas including limbic areas [120]. If FER impairment after STN DBS surgery had been exclusively due to levodopa reduction, the levodopa equivalent dose (LED) reduction should have been more pronounced in those studies showing a substantial FER impairment after DBS, which was not the case (LED reduction ranging from 10 to 76%) [40–42]. Vice versa, the studies that found no significant FER differences should have had small LED reduction, which was again not the case (ranging from 19 to 63%) [63, 64]. Peron et al. [41] showed a postoperative FER deficit of fear and sadness irrespective of dopaminergic medication modification, and Enrici et al. [48] found no correlation of FER with LED in the two PD groups (PD group on dopaminergic therapy and PD group under STN DBS and dopaminergic therapy). On the other hand, Mondillon et al. [62] showed a greater benefit in FER performance when the two therapies (DBS and L-Dopa) were combined. Moreover, another study [121] found that levodopa reduced the reaction time in both the facial emotional and control Stroop subtasks in PD patients postoperatively. Another study [122], using an emotional valence-dependent categorization task a few days after surgery with the stimulator not yet turned on, showed that dopamine enhanced processing of pleasant information.

In studies assessing the ON versus OFF stimulation condition, while there was a worse FER performance ON stimulation and off medication in some studies [43, 62], in other studies [44, 46], there was no significant FER impairment on medication. Nonetheless, even in the studies that tested patients on medication [40–42, 44, 46–49, 63], it is unclear if it was the “best on” due to potential dopaminergic fluctuations [73]. Moreover, the patients were not under their regular medication in all cases (for example, Mondillon et al. [62] defined as on medication the situation 1 hour after the intake of 1.5 of the usual morning L-dopa dose). On the other hand, off medication was defined as being off medication for 12 [43, 46, 62] or 24 hours [49]. Based on these results, it is plausible to hypothesize that impaired FER after DBS is unlikely to be explained by a sole dopamine deficiency but L-dopa might interfere subtly with DBS effects and compensate the FER worsening to an extent. Indeed, controlled L-dopa doses may partially correct the stimulation-induced inactivation of the orbitofrontal cortex and activate the striatocortical circuit [62]. Additionally, dopamine modulates the activity of glutamatergic cortical and GABAergic pallidal afferents to the STN [88]. Moreover, both STN DBS and dopaminergic treatment reduce the pathological increase in beta oscillations [123–125], induce functional inhibition of the STN, and have synergistic effects (the so-called hyperdopaminergic behavioural effects) [27].

Whereas much attention has been directed to the role of dopamine in emotional processing in PD, another issue to be addressed is the role of other neurotransmitters. There is evidence that serotonin plays a role in emotional processing from facial stimuli [126–128] and can modulate the basal ganglia circuitry [129]. Indeed, the basal ganglia including the STN receive serotoninergic innervation from the raphe nuclei [130]. Thus, the behavioural effects of DBS could be induced by the interaction between STN and midbrain raphe serotonergic neurons [131]. Indeed, bilateral high-frequency stimulation of the STN inhibited the firing rate of serotonergic neurons in the dorsal raphe nucleus [132] and serotonin release in the prefrontal cortex and hippocampus in animal PD models [133]. Moreover, apart from serotonergic, noradrenergic systems seem to play a role in the STN DBS effects as well [134]. It is possible that different functions within the STN are mediated by different neurotransmission systems and that distinct but overlapping neuronal populations modulate STN output [86]. High-frequency stimulation reduces STN hyperactivity and, apart from restoring the function of the dopaminergic system in the motor territories, may disturb the balance between the dopaminergic and other neurotransmission systems [40, 86].

### 5.4. Contribution of Cognitive and Other Neuropsychiatric Symptoms to Facial Emotion Recognition after STN DBS

Emotions are closely related to cognitive processes and are often determined by the cognitive evaluation of events, depending on the meaning of these events for the individual's welfare and goals [73]. In fact, the identification of emotions can be seen as a complex cognitive process, relying on many cognitive domains such as working memory, language, and visuospatial perception [78]. Most of the studies assessing FER after DBS measured neuropsychological function as well [40–42, 44, 46–48, 63]. Regarding the contribution of cognitive changes to FER worsening after DBS, while some studies, which showed a total or specific emotion FER worsening after DBS also showed worsening of cognitive measures such as verbal fluency [40, 41] or correlation between the two functions [46], others did not [41, 42, 63]. Moreover, most studies did not find a connection between FER worsening after DBS and global cognitive measures [40, 42, 44, 63] or executive functions [41, 44, 63], which remained unchanged after surgery. On the contrary, studies that did not find FER impairment after STN DBS reported a significant improvement in some neuropsychological measures such as mini mental state examination and immediate recall [46, 47]. It is also noteworthy that the different tasks assessing emotion recognition vary in the cognitive resources they demand [52]. The contribution of visuospatial perception decline after surgery to FER worsening is also a controversial issue as some studies reported a worsening of visuospatial abilities postoperative [135, 136], whereas others [40] found a FER impairment without a visuospatial perception deficit. It is noteworthy that not all studies did a nonemotional facial recognition test such as the Benton test (although such deficits are not common in PD patients) and only two studies [43, 62] tested for visual contrast sensitivity. Visual and emotional systems are indeed closely connected: the amygdala is connected to superior colliculus, anterior cingulate, orbitofrontal, and cortical temporal visual areas [137], but it seems unlikely that the complex emotional recognition process is solely dependent on the visual perception abilities, which participate in the rather early stages of FER [79].

A common neuropsychiatric effect of STN DBS is the modulation of inhibitory control [138]. STN DBS can alter impulse control and in some cases induce or exacerbate certain impulsive behaviour in PD patients [139]. The inhibition as a cognitive process is essential to emotional processing [73]. Indeed, the inhibitory (no-go) signal from the STN, mediated by connections with frontal areas [138], delays automatic responses and gives additional time for central processing of a behaviour [140]. From another perspective, it could be assumed that the worsening in FER after DBS could be partly due to impairment of inhibition control leading to more impulsive decisions and inaccurate choices of the right emotion. In that case, reaction times after presentation of facial emotional stimuli would be shorter in the ON condition, similar to the global decrease in reaction time in response to high conflict trials [140, 141]. The majority of studies did not assess reaction times for FER tasks. A study [121] using an emotional Stroop task showed that stimulation (ON condition) significantly reduced reaction times, whereas another [45] showed longer reaction times specifically for disgust recognition irrespective of stimulation condition. The potential involvement of anxiety, depression, or apathy in FER impairment after DBS is another issue not widely addressed among studies possibly because patients with major affective disturbances were excluded preoperatively. Nevertheless, FER impairment in PD occurs independently of patients' depression status [76]. Interestingly, Dujardin et al. [40], who found a worsening of FER, found a reduction of anxiety after surgery. In the study of Albuquerque et al. [47], the neuropsychiatric symptoms (apathy and depression) could not be predicted from the emotion recognition tests. Moreover, Drapier et al. [63] found no correlation between the postoperative worsening of apathy and emotion recognition and suggested that each of these functions has separate functional networks, probably passing through the STN. On the other hand, Enrici et al. [48] found a significant negative correlation between apathy and FER performance in both PD groups (receiving dopaminergic therapy or both dopaminergic therapy and STN DBS).

### 5.5. Neurosurgical Issues

The neurosurgical target for DBS in PD is the sensorimotor area of the STN (dorsolateral territory). However, the small size of this structure (approximately 3 mm coronal × 6 mm sagittal × 12 mm axial) compared with the size of each contact of the implanted electrode (1.5 mm high × 1.27 mm wide) suggests that DBS may influence other areas of the STN besides the motor one and particularly its limbic territory, through current diffusion depending on pulse width and voltage [40]. Moreover, there seems to be a substantial overlap between the different areas of the STN [13] and there is evidence that they are connected by GABAergic interneurons [142]. Indeed, Lambert et al. [89] reported that most cortical regions had projections to all the STN functional subterritories and vice versa. Another factor is the role of surgical trajectory for the electrode placement: electrodes are inserted through the frontal lobes (and possibly the dorsolateral prefrontal cortex) and often cause lesion of fibres connecting the thalamus or the head of the caudate nucleus with the frontal lobes, which are regions involved in higher cognitive processes [36]. Indeed, York et al. [143] observed that cognitive and emotional changes six months following bilateral STN DBS may be related to the surgical trajectory and electrode placement. The implantation of the electrode might also affect different cognitive functions such as attention and working memory [33], as well as patients' performance in emotion recognition tasks by increasing impulsivity [138]. There is also a “microlesion” effect, which reflects the posttraumatic tissue reaction within the STN caused by the implantation of electrodes [144]. This effect, although typically short lived and less likely to affect the DBS outcome, can induce changes to the regional metabolism in STN, globus pallidus, ventral thalamus, and sensorimotor cortex [145, 146].

### 5.6. Lateralization

The connections between STN and cortex are ipsilateral [89]. Emotional auditory stimuli evoked activity in the right ventral STN in an electrophysiological study [147]. Another study [121] reached the conclusion that STN DBS induced hypoactivation of the right fusiform gyrus. Moreover, an imaging study [66] showed that the inhibition of the activity of the lateral fusiform face area was the result of the stimulation of the right STN. In another neuroimaging study [107], there was an asymmetry found in a patient with DBS-induced hypomanic episodes, with the left STN showing lower connectivity to the prefrontal cortex. Additionally, Lambert et al. [89] reported partly asymmetrical projections of the STN with the temporal pole favoring the left and the orbital gyrus favoring the right. All limbic connections were more prominent in the left hemisphere apart from a right-sided dominance of connections with the middle-frontal gyrus, middle anterior cingulate, and superior precentral gyrus [89]. Thus, there might be a lateralization favoring the right STN, in accordance with the knowledge that the right hemisphere is generally more active in emotional processing [148]. Interestingly, Coundouris et al. [56] in their meta-analysis found that patients with left side PD onset, i.e., right hemisphere-driven pathology had poorer emotion recognition ability. As most studies did not examine this parameter, future research could investigate the effect of variable stimulation of the right STN on social abilities or even inactivation in specific (emotional demanding) social situations [66].

### 5.7. Impact of STN DBS on Specific Emotions

Regarding misattribution of emotions, Biseul et al. [44] found that most common misattribution of fear in the postoperative PD group was surprise, while Peron et al. [41] reported that the pattern of misattribution did not change as compared to before surgery. The misattribution for negative emotions could be due to various reasons. Negative emotions are generally more difficult to recognize [149, 150] having overlapping features unlike happiness that can be easily recognized from the feature of smile [151, 152]. From another perspective, it could be due to the general increase in positive affect, which has been linked to STN DBS [96, 153]. It seems that some neural areas are engaged in the perception of all basic emotions such as the amygdala, the ventral striatum, and frontal and temporal areas [154–156] but the activation pattern of recognition of separate emotions is partially distinct [154]. Additionally, one neural structure can have multiple functions, depending on the functional network and coactivation pattern at a given moment [155]. Another reason could be that the areas associated with the recognition of negative emotions may be subject to greater dopaminergic denervation in PD such as the amygdala, insula, and the orbitofrontal and anterior cingulate cortices [157–159] or that they are involved in an archaic evolutionary preserved route responsible for the recognition of threatening stimuli which might be affected in PD [160]. However, whether STN or its subareas are particularly associated with the network processing negative emotions is not clear. Le Jeune et al. [42] suggested that the negative emotion network passes through the STN, whereas Peron et al. [73] proposed that STN DBS induces modifications in all components of emotion irrespective of stimulus valence (positive or negative). As happiness was the only positive emotion tested across studies (surprise can be viewed as a transition emotion) and the anatomical substrates for positive emotions are much less investigated (with the exception of superior temporal gyrus and anterior cingulate cortex for the processing of happiness [156, 161]), future studies should include more positive emotions (e.g., gratitude, serenity, hope, pride, amusement, inspiration, and relief) as well as more complex negative emotions (e.g., annoyance, anxiety, guilt, despair, and jealousy).

### 5.8. Are There Risk Factors for Facial Emotion Recognition Changes after STN DBS?

It seems that different risk factors such as patients' vulnerability before DBS, dopamine dosage, or stimulation [37] may influence the STN DBS neuropsychiatric outcome. Indeed, patients with marginal cognitive or behavioural functioning such as older patients are at risk of developing postoperative behavioural decompensation [162]. Other factors that could explain why such behavioural symptoms differ between patients after surgery could be personality traits, the social environment, cultural differences, and learned behaviours [36]. The anatomical variability between subjects [107] and the variability in terms of cognitive capacities (e.g., mild cognitive impairment) should also be taken into consideration. Another aspect that could be explored in future studies is whether FER worsening after DBS occurs in a subgroup of patients with distinct nonmotor characteristics, i.e., a predominant nonmotor subtype for example the nontremor dominant subgroup, which is more associated with cognitive and affective symptoms [120], or the diffuse phenotype, likely to have mild cognitive impairment, orthostatic hypotension, and REM sleep behaviour disorder at baseline and more rapid progression of nonmotor symptoms [163]. Argaud et al. [52] suggested that hypomimia may play a role to emotional processing difficulties in PD. Thus, a subject of future studies could also be to examine hypomimia after STN DBS in relation to FER change. Therefore, there seems to be a complex interplay between predisposition, surgical, and postoperative issues.

## 6. Conclusion

In summary, the majority of studies published so far showed that facial emotion recognition in PD patients after STN DBS surgery worsens compared to the condition before surgery [40–42, 63], while a few studies showed no significant impairment of FER after STN DBS [46, 64]. In addition, studies showed worse FER in the ON STN condition compared to OFF without dopaminergic medication [43, 62], while on medication there was no significant difference reported [46, 62]. The main findings and considerations regarding the effects of STN DBS on FER are summarized in Table 2. Limitations of the current review should be acknowledged such as the small sample sizes of studies, the variable follow-up periods after surgery, and possibly different sensitivity of FER testing used among studies, as well as the fact that the studies were mainly observational and not randomized control trials. Moreover, it cannot be excluded that studies with positive findings were more likely to be published compared to studies showing no difference after DBS. Additionally, the studies were conducted in PD patients, where the STN involvement might reflect a compensatory response. Nevertheless, evidence points to a functional role of STN in limbic circuits. Indeed, there are various factors that need to be elucidated in future studies such as the methodological discrepancies of studies, neurosurgical issues, the role of the disease itself, and that of dopaminergic medication. Whether the postoperative FER changes are transient or persistent is also unclear at the moment. Therefore, long-term follow-up studies with testing at various time points after surgery are needed. Moreover, larger patient cohorts should be tested in future studies using standardized, validated neuropsychological measures of FER, which would include all basic emotions and measure both FER response accuracy and reaction time as outcome. Furthermore, it would be interesting for future studies to look at the correlation of FER outcomes with electrode position in relation to STN and volume of tissue activated by DBS.

FER changes after STN DBS can be attributed to the functional role of the STN in cognitive and limbic circuits [103, 164, 165] or to the interference of STN stimulation with the integration of neural networks involved in FER [42, 66]. Importantly, networks are not static but dynamic [166], adapting to current demanding tasks or situations. In this way, FER might be affected variably in the time course after DBS. Thus, the role of STN is extended: STN represents a central position for multilevel integration of motor, cognitive, and affective information [107]. DBS interferes with information interplay in the STN, coming from structures such as the prefrontal cortex, anterior cingulate, and amygdala. STN stimulation facilitates the recruitment of movement-related prefrontal areas, which is accompanied by motor improvement [167, 168]; however, it might exert an opposite effect on associative and limbic basal ganglia projection areas and lead to inflexibility of mental responses [169]. Hence, STN high-frequency stimulation is capable to restore the motor circuit, but might cause a functional imbalance in the nonmotor (limbic) circuit, which could explain why most studies reported worsening of FER after DBS.

Facial expressions are strong nonverbal displays of emotions, which signal valence information to others and are important communication elements in social interactions. Future studies should assess if difficulties in emotion recognition and processing have an impact on patients' and caregivers' quality of life. Indeed, patients after DBS surgery exhibit frequently difficulties in their relationship with close family members and their socioprofessional environment [170]. Impaired FER might contribute to these difficulties in interpreting social cues. Another issue is whether the neuropsychiatric deficits after DBS could be improved through interventional strategies or even prevented. This stresses the importance of neuropsychological approach of PD patients after STN DBS, favorably in the context of a multidisciplinary team, in order to optimize motor and nonmotor DBS outcome.

DBS is an effective therapy for PD. There is plenty of evidence that it is more effective than optimal drug therapy [24]. Carefully selected patients experience besides a significant motor improvement a substantial benefit in the quality of life [23], which outlasts adverse effects. DBS is an important therapeutic intervention for patients with medically intractable motor symptoms, in whom nonmotor symptoms are not predominant [1, 24], which stresses the importance of individualization of PD treatment depending on patients' symptoms. The aspects discussed in the present article improve our understanding of the role of the STN in emotional control in the growing field of affective neuroscience. However, the impact of STN DBS on social perception abilities requires further research. Carefully designed studies in PD patients prior to and after STN DBS can add to our knowledge concerning the role of STN in social interaction and better inform individualized clinical decisions on DBS treatment in PD.

## Figures and Tables

**Table 1:** Studies accessing facial emotion recognition after STN DBS in PD.

Study	Number of participants (m, f)	FER test stimuli (number of stimuli and emotions, display time)	Age (years)	Disease duration (years)	Hoehn and \Yahr score (pre-DBS)	LED mean (mg/day) pre-DBS//post-DBS	Assessment conditions	Assessment time mean ± sd (range)	Outcome
Schroeder et al. 2004 [43]	10 PD (6m, 4f)	4 blocks each of 60 computer-transformed facial stimuli from the Ekman and Friesen series, 6 emotions, each stimulus containing different intensities of two emotions, display time n/a	61 ± 11.1	16 ± 3.1	n/a	n/a//600	ON STN vs. OFF STN (med off)	11 ± 7 months (3-24) after DBS	Anger recognition accuracy significantly reduced in the ON STN condition

Dujardin et al. 2004 [40]	12 PD (5m, 7f) 12 HC	12 facial stimuli from Hess and Blairy series, 2 (30% and 70% expression intensities) × 3 emotions (anger, disgust, sadness) × 2 (male and female), more than 3 sec	57.5 ± 6.5	13 ± 2.5	4 (3-5) off med state	1472 ± 510//777 ± 323	Pre vs. post op (med on vs. med on, STN ON)	Before and 3 months after DBS	Sign. reduction of total FER accuracy, sadness, and anger, (trend for disgust) regardless of expression intensity

Biseul et al. 2005 [44]	Different PD patients before and after DBS (15 (9m, 6f) in each group, matched for disease duration) 15 HC	55 facial stimuli from Ekman and Friesen series, 7 emotions, 3 s	61.7 ± 8.2 years (post-DBS group)	15 ± 6.2 years (post-DBS group)	n/a	n/a	Pre vs. post DBS ON vs. OFF STN (on med in all conditions)	Before and 7.2 ± 12.1 months after DBS (1-48 months)	Pre- vs. post-DBS: sign. reduction of fear accuracy (either ON or OFF STN) post-DBS ON vs. OFF STN: no sign. difference for all emotions

Geday et al. 2006 [66]	7 PD 22 HC	6 series each of 30 facial stimuli from the Empathy Picture System, 3 emotions (sadness, neutral, happiness), 3 sec	61.1 ± 9.1 years	n/a	n/a	n/a	ON vs. OFF STN DBS (med off)	3-25 months after DBS	Faces were scored as less pleasant ON DBS in comparison to OFF (rating on a scale from -3 to +3)

Drapier et al. 2008 [63]	17 PD (11m, 6f)	55 facial stimuli from Ekman and Friesen series, 7 emotions, 3 sec	56.9 ± 8.7	11.8 ± 2.6	0.88 ± 0.5 (on med)	1448 ± 400//1175 ± 443	Pre- vs. post-op (med on vs. med on, STN ON)	3 months before and 3 months after DBS	Sign. reduction in the recognition accuracy of fear and sadness

Le Jeune et al. 2008 [42]	13 PD (9m, 4f) 30 HC	55 facial stimuli from Ekman and Friesen, 7 emotions, 3 sec	57 ± 7.8	10.9 ± 2.2	1 ± 0.6 (on med)	1066.2 ± 347//957.3 ± 494.6	Pre- vs. post-op (on med vs. on med, ON STN)	3 months before and 3 months after	Sign. reduction of total FER and fear score

Peron et al. 2010 [41]	24 PD STN DBS (17 m, 7f), 20 treated with apomorphine (APO), 30 HC	55 facial stimuli from Ekman and Friesen, 7 emotions, 3 sec	59 ± 8	11.9 ± 2.5	1.0 ± 0.6 (on med)	1307 ± 338//987 ± 406	Pre vs. post (med on vs. med on, STN ON)	3 months before and 3 months after DBS	Sign. reduction of total FER accuracy, sadness, fear after DBS

Mondillon et al. 2012 [62]	14 PD (9m, 5f) 14 HC	56 facial stimuli in each block from Karolinska directed emotional faces database, 7 emotions, 500 ms	60.57 ± 1.64	12.36 ± 0.71	n/a	post-DBS 1042.5 ± 106.97	4 conditions post-op (med off, STN OFF; med off, STN ON; med on, STN ON; med on, STN OFF)	At least 6 months after (3.5 ± 0.5 year)	ON vs. OFF STN DBS (off med): sign. worse recognition accuracy in ON condition ON vs. OFF STN DBS (on med): better FER recognition accuracy in ON condition *Greater FER benefit when two therapies (L-Dopa, DBS) combined*

Aiello et al. 2014 [46]	12 PD (8m, 4f) 13 HC	30 facial stimuli from Nim Stim Set, 6 emotions, display time n/a	61.7 ± 7.4	10.9 ± 4.1	n/a	n/a	Pre- (on- and off- medication) vs. post-DBS (on med, OFF STN and on med, ON STN)	Before and after DBS: OFF STN: 5 days after ON STN: 2-6 months after	Pre- vs. post-DBS (on med vs. on med, ON STN): no sign. FER accuracy difference Pre- vs. post-DBS (on med vs. on med, OFF STN): no sign. FER accuracy difference ON vs. OFF stimulation (on med): no sign. FER accuracy difference

Albuquerque et al. 2014 [47]	30 PD (18m, 12f)	16 facial stimuli from CATS, 7 emotions, no time limits	62.7 ± 7.7	15.85 ± 7.02	2.21 ± 0.25 (on med)	1148 ± 433.5//425 ± 209	Pre vs. post-op (on med vs. on med, STN ON)	Before DBS and 1 year after	No sign. accuracy difference in FER tasks (neither for positive nor for negative emotions)

Mermillod et al. 2014 [65]	14 PD (9m, 5f) 14 HC	105 facial stimuli from the Ekman and Friesen series in broad (BSF), high (HSF > 24 cycles/image) and low (LSF < 8 cycles/image) spatial frequency resolutions, 7 emotions, 200 ms	60.57 ± 1.64	12.36 ± 0.71	n/a	post-DBS 1042.5 ± 106.97	Post-DBS (4 conditions: med off, STN OFF; med on, STN OFF; med off, STN ON; med on, STN ON)	At least 6 months after DBS (3.5 ± 0.5 years)	ON vs. OFF: no sign. effect of stimulation for BSF and LSF faces, lower total FER accuracy for HSF in the ON condition

McIntosh et al. 2015 [49]	Two early PD groups: 7 PD (5m, 2f) optimal drug therapy, 9 PD (8m, 1f) optimal drug therapy and STN DBS, 21 matched young and 23 aged HC	Facial emotional stimuli from TASIT (EET part, 28 stimuli) and RMET test (36 pictures), complex emotions, display time n/a	62.22 ± 7.97 (optimal drug therapy+DBS group)	n/a	≤2	348.7 ± 240.3 (optimal drug therapy + DBS group)	ON condition∗ = optimal drug therapy and optimal DBS OFF condition∗ = off med (24 h) and OFF DBS (24 h)	n/a	No accuracy difference between the PD groups (optimal drug therapy or optimal drug therapy and DBS) or treatment conditions (ON∗, OFF∗)

Enrici et al. 2017 [48]	18 PD (9m, 9f) STN DBS 20 PD receiving DRT 20 HC	60 pictures of Ekman test, 6 basic emotions, display time n/a	60.89 ± 6.26 (STN-DBS group)	12.56 ± 3.03 (STN-DBS group)	2.06 ± 1.08 (on med)	STN-DBS group: 760.44 ± 384.29 DRT-PD group: 1074.45 ± 431.6	On med (DRT-PD group) on med, ON STN (STN-DBS group)	1.72 (±1.18) years	No statistically sign. FER accuracy differences between the DRT-PD and STN-DBS groups

Wagenbreth et al. 2019 [45]	14 PD (10m, 4f)	Implicit and explicit emotional processing task, regions around the eyes from the Ekman 60 faces test, 112 stimuli (16 faces, 96 words), 4 emotions (happiness, fear, disgust, neutral), no time limit	61.9 ± 11.46	11.71 ± 4.46^†^	n/a	Post-DBS 386.79 ± 263.76^†^	On med, ON STN vs. OFF med, OFF STN	20.86 ± 27.14^†^ months after DBS (3-77 months)	STN-DBS ON vs. OFF: for the explicit emotional processing task in the ON condition general decrease in response accuracy, sign. decrease in accuracy and longer reaction time for disgust, improved accuracy for fear

Abbreviations: STN: subthalamic nucleus; DBS: deep brain stimulation; FER: facial emotion recognition; PD: Parkinson's disease; HC: healthy controls; sd: standard deviation; n/a: not available; m: male; f: female; 7 emotions: happiness, sadness, fear, surprise, disgust, anger, and no emotion; ms: milliseconds; LED: levodopa equivalent dose; vs.: versus; sign.: significant; med: medication; ON STN: on stimulation; OFF STN: off stimulation; DRT: dopamine replacement therapy; CATS: comprehensive affect testing system; TASIT: Awareness of Social Inference Test; EET: Emotion Evaluation Test; RMET: reading the mind in the eye task; BSF: broad spatial frequency; HSF: high spatial frequency; LSF: low spatial frequency. ^†^Calculated from reported data. Note: the outcome refers to comparisons of PD patients' different conditions (not comparisons with HC).

**Table 2:** Main findings and considerations regarding the effects of STN DBS on facial emotion recognition (FER) in PD.

(i) The majority of studies, which had clinical and methodological heterogeneity, showed that FER in PD patients worsens after STN DBS compared to before surgery, particularly for negative emotions (sadness, fear, anger, and tendency for disgust).
(ii) Most studies showed worse FER in the ON STN condition compared to OFF without dopaminergic medication, while on medication there was no significant difference.
(iii) Neurophysiological studies showed modulation of STN alpha activity after DBS in response to emotionally arousing pictures.
(iv) Neuroimaging studies showed changes in glucose metabolism or regional blood flow after STN DBS in areas associated with FER such as the orbitofrontal cortex, the anterior cingulate cortex, the fusiform gyrus, or amygdala.
(v) Impaired FER after STN DBS is unlikely to be explained by a sole dopamine deficiency resulting from dose reduction postoperatively, but L-dopa might subtly interfere with DBS effects and compensate FER worsening to an extent.
(vi) An association between FER worsening after DBS and global cognitive measures could not be shown in most studies, while the contribution of visuospatial abilities postoperative is a controversial issue.
(vii) FER worsening might be associated with impairment of inhibition control after STN DBS, whereas the potential involvement of anxiety, depression, or apathy is unclear at the moment.
(viii) The surgical trajectory, electrode positioning, stimulation, and current diffusion to nearby limbic STN territory might contribute to DBS effects on FER.
(ix) FER worsening after STN DBS can be attributed to the functional role of the STN in limbic circuits and the interference of STN stimulation with neural networks involved in FER such as the connections of the STN with the limbic part of the basal ganglia and pre- and frontal areas.
